# Endoplasmic reticulum stress downregulates PGC-1α in skeletal muscle through ATF4 and an mTOR-mediated reduction of CRTC2

**DOI:** 10.1186/s12964-022-00865-9

**Published:** 2022-04-15

**Authors:** Marta Montori-Grau, David Aguilar-Recarte, Mohammad Zarei, Javier Pizarro-Delgado, Xavier Palomer, Manuel Vázquez-Carrera

**Affiliations:** 1 Department of Pharmacology, Toxicology and Therapeutic Chemistry, Faculty of Pharmacy and Food Sciences and Institute of Biomedicine of the University of Barcelona (IBUB), University of Barcelona, Barcelona, Spain; 2 Spanish Biomedical Research Center in Diabetes and Associated Metabolic Diseases (CIBERDEM), Instituto de Salud Carlos III, Madrid, Spain; 3 Pediatric Research Institute-Hospital Sant Joan de Déu, Esplugues de Llobregat, Spain; 4 Department of Biochemistry and Molecular Biomedicine, Faculty of Biology, University of Barcelona, Barcelona, Spain; 5 John B. Little Center for Radiation Sciences, Harvard T.H. Chan School of Public Health, Boston, USA; 6 Unitat de Farmacologia, Facultat de Farmàcia i Ciències de l’Alimentació, Av. Joan XXIII 27-31, 08028 Barcelona, Spain

**Keywords:** ER stress, PGC-1α, mTOR, CRTC2, IRS1, Skeletal muscle

## Abstract

**Background:**

Peroxisome proliferator-activated receptor γ (PPARγ) coactivator 1α (PGC-1α) downregulation in skeletal muscle contributes to insulin resistance and type 2 diabetes mellitus. Here, we examined the effects of endoplasmic reticulum (ER) stress on PGC-1α levels in muscle and the potential mechanisms involved.

**Methods:**

The human skeletal muscle cell line LHCN-M2 and mice exposed to different inducers of ER stress were used.

**Results:**

Palmitate- or tunicamycin-induced ER stress resulted in PGC-1α downregulation and enhanced expression of activating transcription factor 4 (ATF4) in human myotubes and mouse skeletal muscle. Overexpression of ATF4 decreased basal PCG-1α expression, whereas ATF4 knockdown abrogated the reduction of PCG-1α caused by tunicamycin in myotubes. ER stress induction also activated mammalian target of rapamycin (mTOR) in myotubes and reduced the nuclear levels of cAMP response element-binding protein (CREB)-regulated transcription co-activator 2 (CRTC2), a positive modulator of PGC-1α transcription. The mTOR inhibitor torin 1 restored PCG-1α and CRTC2 protein levels. Moreover, siRNA against S6 kinase, an mTORC1 downstream target, prevented the reduction in the expression of CRTC2 and PGC-1α caused by the ER stressor tunicamycin.

**Conclusions:**

Collectively, these findings demonstrate that ATF4 and the mTOR-CRTC2 axis regulates PGC-1α transcription under ER stress conditions in skeletal muscle, suggesting that its inhibition might be a therapeutic target for insulin resistant states.

**Video Abstract**

**Supplementary Information:**

The online version contains supplementary material available at 10.1186/s12964-022-00865-9.

## Background

The underlying causes responsible for the development of insulin resistance and its progression to type 2 diabetes mellitus (T2DM) are numerous, but several pieces of evidence indicate that endoplasmic reticulum (ER) stress plays an important role [1]. The main functions of the ER are the synthesis, folding and transport of proteins. Several stimuli, including elevated levels of plasma saturated free fatty acids [2], can disrupt homeostasis in this organelle, leading to ER stress. This results in the activation of an adaptive unfolded protein response (UPR), which restores ER folding capacity and attenuates stress [1]. The UPR involves the activation of three transmembrane proteins: inositol-requiring enzyme 1 (IRE-1), activating transcription factor 6 (ATF6), and protein kinase RNA-like ER kinase (PERK). PERK, once activated, phosphorylates the eukaryotic translation initiation factor 2 (eIF2α) in the α subunit, which results in a minor translation initiation, thereby reducing the load of newly synthesized proteins. Paradoxically, the PERK-eIF2α pathway facilitates the translation of and consequent increase in ATF4 activity, which upregulates the expression of C/EBP homologous protein (CHOP) and other genes.

Skeletal muscle accounts for most of the insulin-stimulated glucose use and, thus, it is the major tissue affected by insulin resistance [3]. Several studies have demonstrated the presence of reduced levels of peroxisome proliferator–activated receptor γ (PPARγ) coactivator 1α (PGC-1α) in skeletal muscle in insulin resistant states [4, 5]. PGC-1α is a master regulator of fatty acid oxidation, mitochondrial biogenesis and reactive oxygen species detoxification. Its downregulation aggravates the inflammatory response and, hence, insulin resistance [6]. However, the role of this transcriptional coactivator in glucose tolerance is complex and it has not been completely clarified, since different genetically modified animal models for *Pgc-1α* have led to different outcomes [7]. Thus, muscle-specific knockout of *Pgc-1α* either reduces [8], improves [9] or does not affect [10] glucose tolerance, while *Pgc-1α* overexpression in skeletal muscle improves glucose tolerance and insulin action [11].

The transcriptional regulation of PGC-1α is mostly controlled by cAMP response element-binding (CREB) protein activation in many tissues [12]. Although it has been reported that ATF4 has a role in the metabolic response to cold stress as a negative regulator of PGC-1α expression in brown adipocytes through the competitive binding with CREB at the *Pgc-1α* promoter [13], little is known about how ER stress regulates PGC-1α in skeletal muscle.

Hyperactivation of mammalian target of rapamycin (mTOR) by nutrient excess has also emerged as a key factor in the development of insulin resistance [14–16]. Interestingly, ER stress increases mTOR activity, whereas mTOR inhibition ameliorates ER stress-induced insulin resistance in myotubes [17]. These findings suggest that mTOR inhibition protects skeletal muscle from ER stress-induced insulin resistance, although the mechanisms involved remain poorly defined.

In the present study, we show that ER stress reduces the levels of PGC-1α in human myotubes and mouse skeletal muscle through a mechanism involving ATF4. In addition, our findings reveal that the activation of mTOR by ER stress inhibits PGC-1α transcription by reducing the nuclear levels of CREB-regulated transcription coactivator 2 (CRTC2) (also known as transducer of regulated CREB activity 2; TORC2), a transcriptional coactivator that greatly enhances CREB-dependent PGC-1α transcription [18–20]. Collectively, our results show that ER stress and mTOR cooperate in myotubes to reduce PGC-1α levels through a new mechanism involving a reduction in CRTC2 nuclear levels.

## Methods

### Reagents

Control, ATF4, tribbles 3 (TRIB3), CRTC2 and S6 kinase (S6K) siRNAs were purchased from Santa Cruz (Dallas, TX).

### Mice

Male C57BL/6 mice (10–12 weeks old) (Envigo, Barcelona, Spain) were housed and maintained under a constant temperature (22 ± 2 °C) and humidity (55%). After 1 week of acclimatization, the mice were randomly distributed into two experimental groups (n = 5 in each): one of the groups was fed a standard diet and the other received a Western-type high-fat diet (HFD, 60% kcal from fat, product D12492, Research Diets Inc., New Brunswick, NJ) for 3 weeks. In the second study, male C57BL/6 mice (10–12 weeks old) were randomly distributed into two experimental groups (n = 5 in each): one of the groups received one intraperitoneal administration of vehicle (DMSO) and the other received one dose of tunicamycin (3 mg/kg) dissolved in the vehicle. Animals were sacrificed 24 h later. In the third study, DMSO (vehicle) (n = 8), tunicamycin (3 mg/kg, n = 4) or tunicamycin (3 mg/kg) and torin 1 (5 mg/kg) (n = 4), were administered to the mice by an intraperitoneal injection before the mice were sacrificed 24 h later. In the fourth study, DMSO (vehicle; n = 8) or torin 1 (5 mg/kg; n = 4) was administered to mice by an intraperitoneal injection. Sixteen hours later, DMSO (control mice; n = 4) or tunicamycin (3 mg/kg; n = 8) was administered and the mice were sacrificed 8 h later. Immediately after sacrifice, skeletal muscle (gastrocnemius) was excised and immediately frozen in liquid nitrogen prior to storage at − 80 °C.

The research complied with the Guide for the Care and Use of Laboratory Animals published by the US National Institutes of Health (8th Edition: National Academies Press; 2011). All experiments involving animals were conducted according to the ethical policies and procedures approved by the Bioethics Committee of the University of Barcelona (Approval no. 228/19). The animals were treated humanely, and all efforts were made to minimize their suffering and the animal numbers. Animal studies were conducted according with the ARRIVE guidelines.

### Cell culture

The LHCN-M2 human skeletal muscle cell line was kindly provided by Dr. W.E. Wright and was cultured and differentiated for 5 days, as previously described [21, 22]. For mammalian cell transfection, LHCN-M2 human myotubes (80–90% confluent) were used 3 days after inducing differentiation. Myotubes grown in 24-well plates (for palmitate oxidation measurement) or 6-well plates (for the other experiments) were transfected with 0.6 µg/well (24-well plates) or 2 µg/well (6-well plates) of pCMV3-ATF4 (Sino Biological Inc., Wayne, PA) or 50 mM siRNA (Santa Cruz Biotechnology Inc., Dallas, TX), using Lipofectamine 2000 (Invitrogen, Carlsbad, CA) in Opti-MEM reduced-serum medium (Life Technologies, Carlsbad, CA). Twenty-four hours post-transfection, cells treatments were conducted as described in the figure legends. pCMV-EGFP-C2 (Clontech, Mountain View, CA) and 50 mM control siRNA-A (#SC-37007, Santa Cruz Biotechnology) were used as the controls for transfection.

### Palmitate oxidation assay in human myotubes

[U-^14^C]-palmitate was used to measure fatty acid oxidation, as previously described [21], except that the cells were grown in 24-well plates in this study.

### RNA extraction, reverse transcription (RT) and real-time PCR

Total RNA from cultured cells was extracted with the Quick-RNA MiniPrep kit (Zymo Research, Irvine, CA), while total RNA from mouse muscle was extracted with TRIsure reagent (Bioline, Memphis TN), following the manufacturer's instructions. 0.5 µg of total RNA were retrotranscribed with TaqMan RT reagents from Applied Biosystems (Foster City, CA) using random hexamers. Real-time PCR was performed in the LightCycler 480 SW 1.5, using the LightCycler 480 Probes Master and probes for the selected human or mouse genes from Applied Biosystems. The 18S rRNA gene was used as the endogenous control to normalize the crossing point (Cp) for each probe assay. The relative gene expression was calculated as 2^−ΔCp^ and the gene fold change was calculated with the 2^−ΔΔCp^ method.

### Western blot analysis

LHCN-M2 cell total protein extracts were prepared by scraping the cell monolayers from 6 cm-diameter dishes or 6-well plates into 100 or 75 µl of homogenization buffer, respectively, containing Ripa Buffer (Sigma Aldrich, St. Louis, MO), 50 mM NaF, 1 mM PMSF, 10 mM Na_3_VO_4_, 10 mM nicotinamide and a protease inhibitor cocktail (Sigma Aldrich). For in vivo studies, 50 mg of mouse muscle were homogenized with a polytron in 500 µl of homogenization buffer. Lysates were then gently rocked for 60 min at 4 °C and centrifuged at 10,000 g for 30 min at 4ºC.

Cytosolic and nuclear fractions were obtained by scraping the cell monolayers from 10 cm-diameter dishes or 50 mg of mouse muscle into 500 µl of homogenization buffer containing 5 mM MgCl_2_, 50 mM Tris–HCl (pH 4.7), 250 mM sucrose and a protease inhibitor cocktail (Sigma Aldrich), as previously described [23].

An aliquot of the supernatant was used to measure protein concentration with the Pierce BCA protein assay kit (Pierce, Thermo Fisher Scientific, Waltham, MA). Proteins were resolved by SDS/10%-(w/v)-PAGE. Immunoblotting was performed with antibodies against PGC-1α (Abcam, Cambridge, UK), α-actinin, histone H2B, CRTC2, ATF4, insulin receptor substrate 1 (IRS1), TRIB3 (Santa Cruz Biotechnology Inc.), and tumour necrosis factor (TNF)-α, total and phospho-S6 ribosomal protein (Ser235/236), insulin receptor β, GAPDH, p44/42 MAPK(ERK1/2), phospho-p44/42 MAPK (Thr202/Tyr204) or phospho-CREB (Ser133), total and phospho-glycogen synthase kinase (GSK)3-α/β (Ser21/9), total and phospho-Akt (Ser473) (Cell Signaling, Danvers, MA), and CRTC2 (Calbiochem, San Diego, CA).

### Statistical analysis

Data are presented as the mean ± SEM. Significant differences were assessed by Student’s t-test or one-way and two-way ANOVA, according to the number of groups compared, using the GraphPad Prism program (V8.4.3) (GraphPad Software Inc., San Diego, CA). The Tukey–Kramer multiple comparison post-hoc test was performed when significant differences were found by ANOVA. Differences were considered significant at *P* < 0.05. The results for gene expression (fold change from the real-time PCR analysis) were examined with the Relative Expression Software Tool (REST).

## Results

### ER stress reduces PGC-1α levels in human myotubes and mouse skeletal muscle

**Fig. 1:**
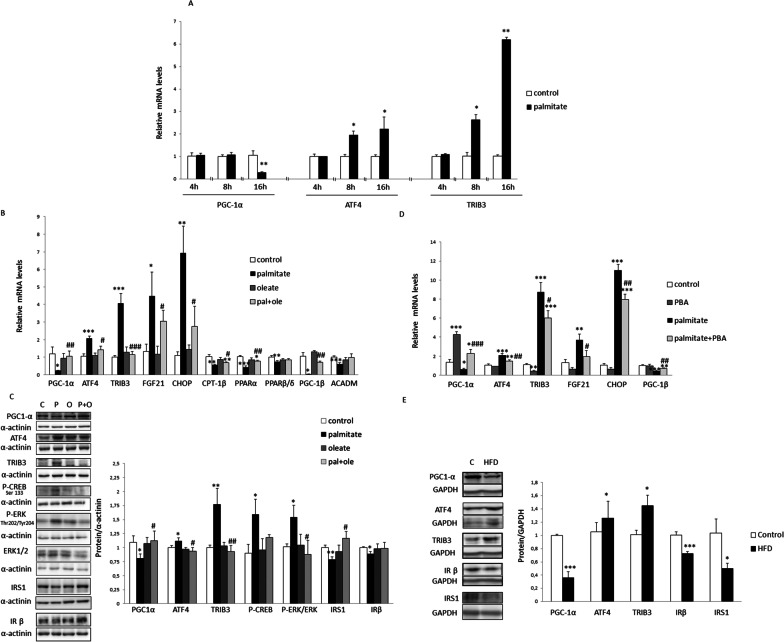
Palmitate-induced ER stress reduces PGC-1α in human myotubes and mouse skeletal muscle. **A** Time course of *PGC-1α*, *ATF4* and *TRIB3* mRNA levels in human LHCN-M2 myotubes incubated in the presence or absence (control) of 0.5 mM palmitate. **B** mRNA or **C** protein levels in human LHCN-M2 myotubes incubated for 16 h in the absence (control) or in the presence of different fatty acids: 0.5 mM palmitate, 0.5 mM oleate or 0.5 mM palmitate supplemented with 0.3 mM oleate. **D** mRNA levels in LHCN-M2 myotubes incubated for 16 h in the absence (control) or presence of 0.5 mM palmitate and/or 2 mM PBA. Data are presented as the mean ± SEM (n = 6–12 per group for mRNA and n = 3–6 for proteins). **E** Protein levels in skeletal muscle of mice fed standard chow or HFD for 3 weeks. Data are presented as the mean ± SEM (n = 5 per group). *p < 0.05, **p < 0.01 and ***p < 0.001 versus control. ^#^*p* < 0.05, ^##^*p* < 0.01 and ^###^*p* < 0.001 versus palmitate-treated cells

First, we assessed the effects of the saturated fatty acid palmitate on PGC-1α expression in human LHCN-M2 myotubes. A time-course study for 4, 8 and 16 h showed that palmitate exposure reduced *PGC-1α* expression only after 16 h (Fig. 1A). This reduction was accompanied by an increase in the mRNA levels of ER stress markers such as *ATF4* and one of its target genes, TRIB3 [24], showing a higher increase at 16 h, thus reflecting the maximum activity of ATF4. Incubation with palmitate for 16 h also increased the expression of additional ATF4-target genes, including FGF21 [25] and *CHOP* (also known as *DDIT3*) (Fig. 1B). PGC-1α acts as a transcriptional coactivator of PPARα to increase the expression of the genes involved in fatty acid oxidation [12]. Consistent with its role in reducing *PGC-1α* expression, palmitate decreased the expression of the genes involved in fatty acid oxidation such as *CPT-1B*, *PPARα*, *PPARβ/δ* and *ACADM* (Fig. 1B). Palmitate also reduced the expression of additional PCG-1 family members, such as *PGC-1β*. In contrast to palmitate, the same concentration of the monounsaturated fatty acid oleate did not induce either ER stress or affect *PGC-1α* expression in the cells. Moreover, co-incubation of the cells with palmitate (0.5 mM) and a lower concentration of oleate (0.3 mM) attenuated or abolished the effects of the former (Fig. 1B), in agreement with the fact that oleate prevents palmitate-induced ER stress [2]. In line with the changes in the mRNA levels, palmitate reduced the protein levels of PGC-1α, and increased those of ATF4 (Fig. 1C). Of note, although PGC1-α was downregulated by palmitate, incubation with this fatty acid led to the activation of CREB as demonstrated by the increase in its phosphorylation at Ser^133^, suggesting the activation of a compensatory mechanism to try to overcome its reduced binding to the *PGC-1α* promoter (Fig. 1C). Palmitate also resulted in a decrease in the protein levels of insulin receptor substrate 1 (IRS1) and the β subunit of the insulin receptor (IRβ). The reduction of the latter is known to be related to the presence of ER stress [26]. ER stress also increases extracellular signal–regulated kinase (ERK)1/2 phosphorylation in myotubes [2], which was consistent with the effect observed following palmitate exposure (Fig. 1C). As observed with the mRNA levels, oleate did not affect the levels of any of these proteins, whereas co-incubation of the myotubes with palmitate and oleate abrogated the effects of the former. Finally, treatment of myotubes with palmitate and the chemical chaperone 4-phenylbutyric acid (PBA) [27], which mitigates ER stress, attenuated or abolished the effects of palmitate, confirming that its effects were mediated by ER stress (Fig. 1D). Consistent with the in vitro studies, HFD-fed mice showed reduced PGC-1α, IRβ and IRS1 protein levels in skeletal muscle, but increased ATF4 and TRIB3 levels (Fig. 1E).

**Fig. 2:**
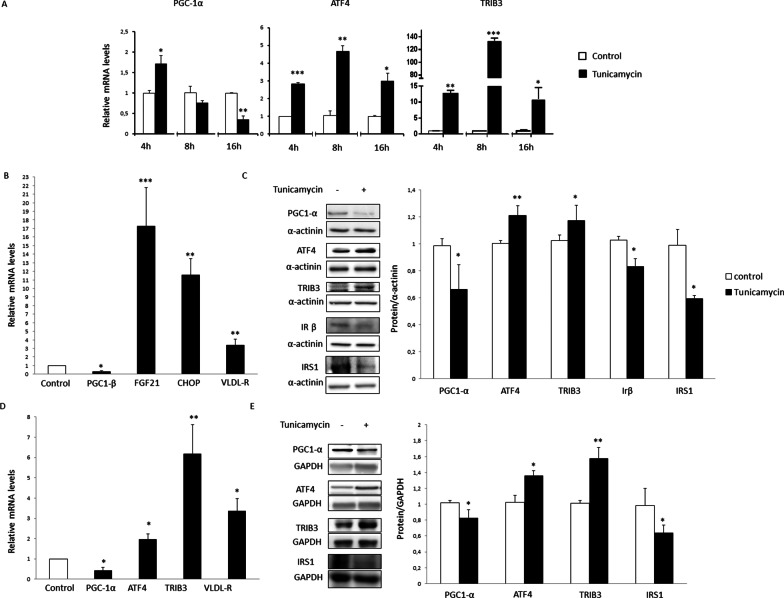
Tunicamycin reduces PGC-1α in human myotubes and mouse skeletal muscle. **A** Time course of *PGC-1α*, *ATF4* and *TRIB3* mRNA levels in human LHCN-M2 myotubes incubated in the absence (control) or presence of 2 µg/ml of tunicamycin. **B** mRNA and **C** protein levels in human LHCN-M2 myotubes incubated for 16 h in the absence (control) or presence of 2 µg/ml of tunicamycin. Data are the mean ± SEM (n = 6–12 per group for mRNA and n = 3–6 for proteins). **D** mRNA and **E** protein levels in the skeletal muscle of mice treated with vehicle or tunicamycin for 24 h. Data are presented as the mean ± SEM (n = 5 per group). *p < 0.05, **p < 0.01 and ***p < 0.001 versus control

To clearly demonstrate that ER stress reduces PGC-1α, we used the ER stressor tunicamycin. A time-course study showed that myotubes exposed to tunicamycin displayed a significant reduction in *PGC-1α* mRNA levels after 16 h, whereas the expression of *ATF4* and *TRIB3* was significantly increased at the three time points analyzed (Fig. 2A). Incubation of myotubes with tunicamycin for 16 h also increased the expression of additional *ATF4*-target genes, including *FGF21*, CHOP and *VLDL-*R [28], but it reduced the expression of *PGC-1β* (Fig. 2B). Induction of ER stress in myotubes also reduced the protein levels of PGC-1α and of those involved in the insulin signaling pathway (IRβ and IRS1), while increasing those of ATF4 and TRIB3 (Fig. 2C). In the skeletal muscle of mice treated with tunicamycin, a reduction was observed in the mRNA levels of *Pgc-1α* that was accompanied by increases in the expression of *Atf4*, *Trib3* and *Vldl-r* (Fig. 2D). The same trend was observed in the protein levels, which was accompanied by a reduction in IRS1 levels (Fig. 2E).

### The ER stress-mediated induction of ATF4 contributes to the reduction of PGC-1α levels in myotubes

**Fig. 3:**
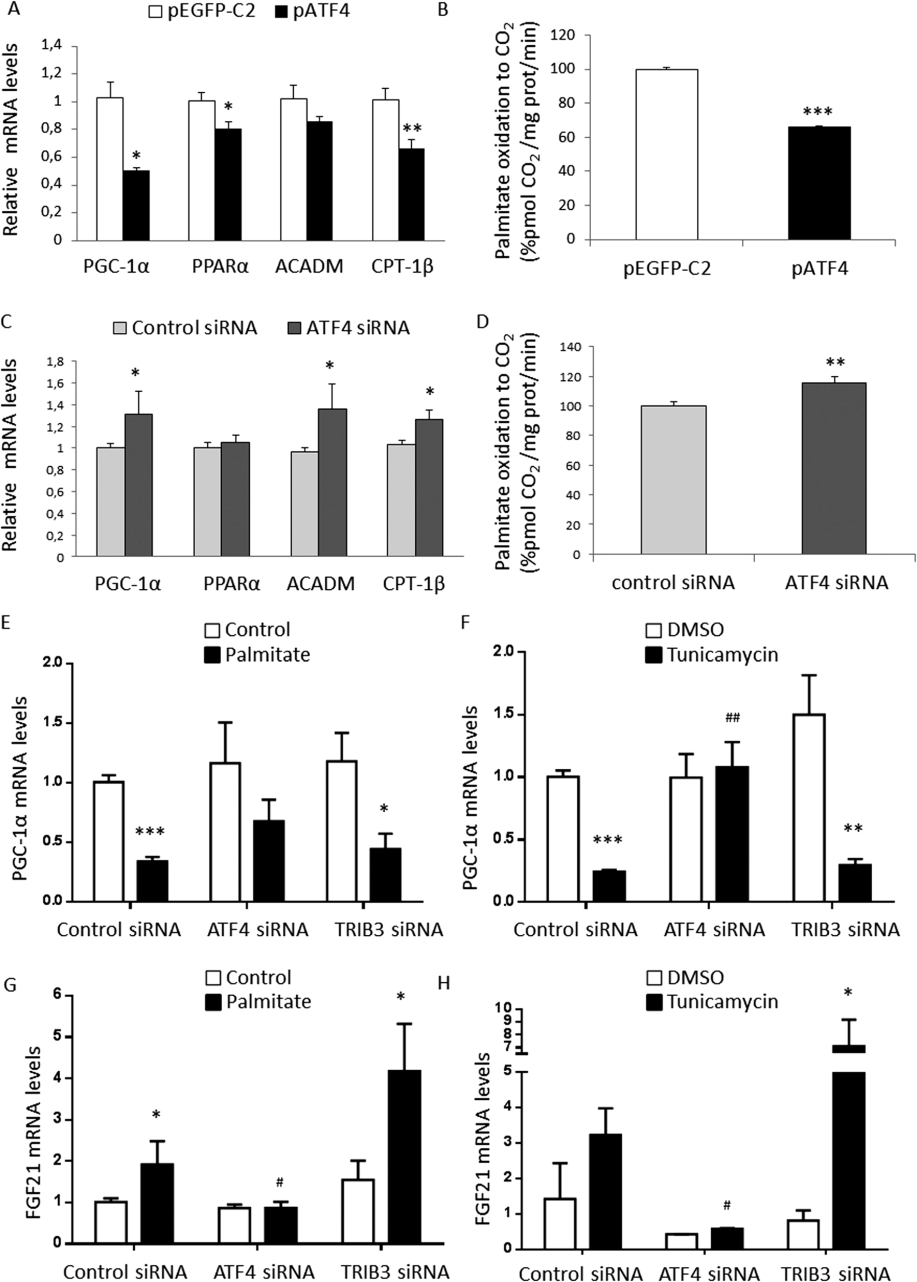
ATF4 is involved in the reduction of PGC-1α caused by ER stress in myotubes. **A** mRNA levels and **B** palmitate oxidation to CO_2_ in LHCN-M2 cultured myotubes transfected with pCMV-GFP-C2 (pGFP-C2) or pCMV-ATF4 (pATF4). Data are presented as the mean ± SEM (n = 6 per group). **C** mRNA and **D** palmitate oxidation to CO_2_ in LHCN-M2 cultured myotubes transfected with control siRNA or ATF4 siRNA. **E**, **F** *PGC-1α* or **G**, **H** *FGF21* mRNA levels in LHCN-M2 cultured myotubes transfected with control, ATF4 or TRIB3 siRNA and incubated with or without (control) 0.5 mM palmitate or 2 µg/ml of tunicamycin for 16 h. Data are presented as the mean ± SEM (n = 3–6 per group). *p < 0.05, **p < 0.01 and ***p < 0.001 versus control. ^#^*p* < 0.05 and ^##^*p* < 0.01 versus control siRNA

Since it has been reported that ATF4 reduces PGC-1α levels in brown adipocytes in the metabolic response to cold stress through competitive binding with CREB at a CRE site in the *Pgc-1α* promoter [14], we explored whether a similar mechanism operates in myotubes during ER stress. Overexpression of ATF4 in myotubes (Additional file 1: Figure S1A) resulted in a reduction in *PGC-1α* expression and a subsequent decrease in the expression of the genes involved in fatty acid oxidation, *PPARα*, *ACADM* and *CPT-1B* (Fig. 3A), although this reduction was not significant for *ACADM*. Consistent with these changes, ATF4 overexpression also reduced fatty acid oxidation (Fig. 3B). To clearly demonstrate that the induction of ATF4 caused by ER stress was responsible for the reduction of PGC-1α in myotubes, we transfected cells with siRNA against this transcription factor. *ATF4* knockdown attenuated both its basal expression and the increase in its expression caused by tunicamycin or palmitate (Additional file 1: Figure S1B). *ATF4* gene silencing upregulated the basal expression of *PGC-1α*, *ACADM* and *CPT-1B* (Fig. 3C), which was consistent with the observed increase in fatty acid oxidation (Fig. 3D). Moreover, *ATF4* knockdown attenuated the reduction in *PGC-1α* expression caused by palmitate (Fig. 3E) and abolished the decrease caused by tunicamycin (Fig. 3F). Knockdown of *TRIB3* (Additional file 1: Figure S1B) had no effect on the reduction of *PGC-1α* expression (Fig. 3E, F), indicating that the effect of *ATF4* gene silencing on *PGC-1α* expression was specific. *ATF4* knockdown also prevented the tunicamycin- and palmitate-mediated induction of its target gene *FGF21* (Fig. 3G, H).

### mTOR inhibition prevents the reduction in PGC-1α expression caused by tunicamycin in myotubes and mouse skeletal muscle

**Fig. 4:**
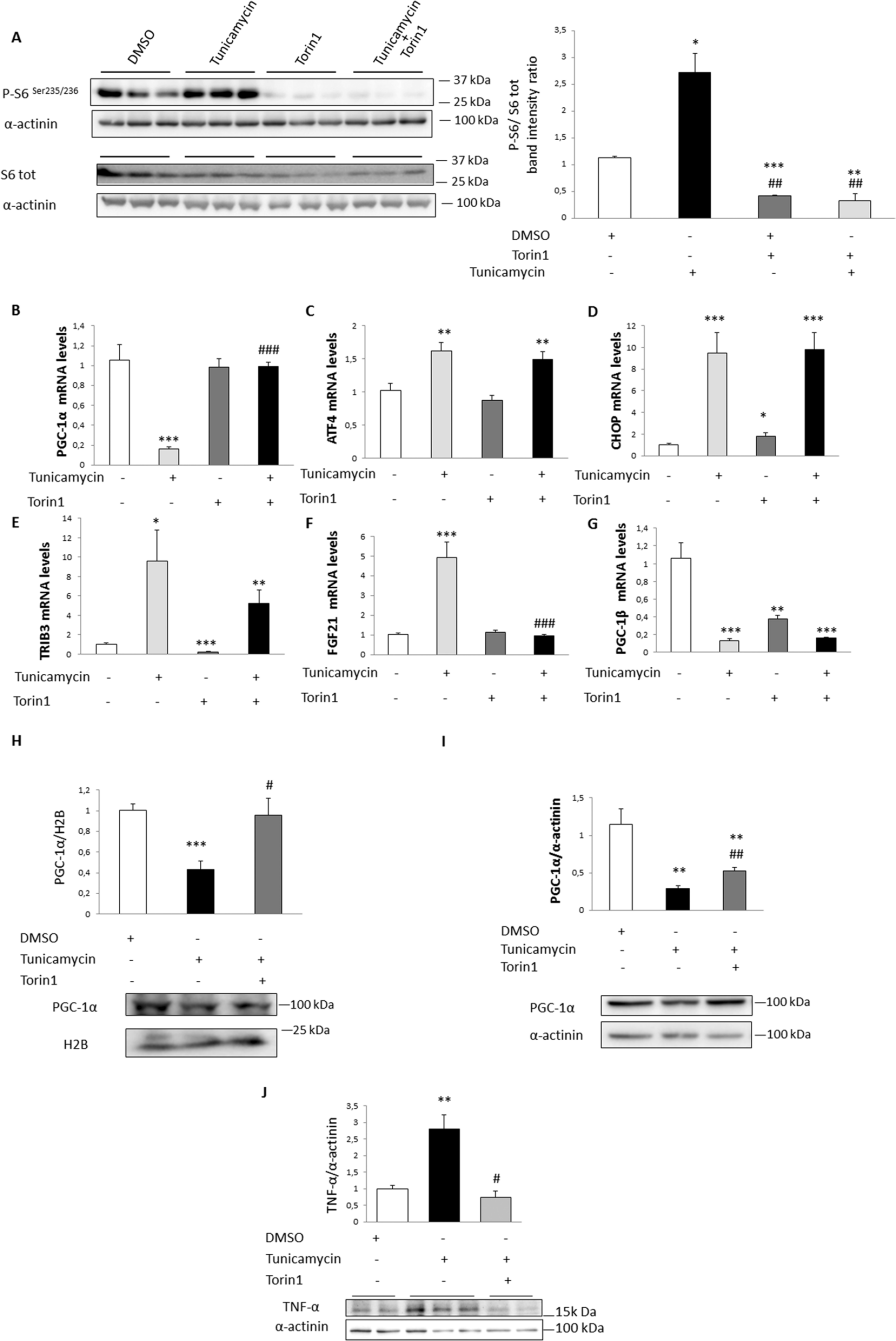
mTOR inhibition prevents ER stress-induced PGC-1α downregulation in myotubes and skeletal muscle. **A** LHCN-M2 myotubes were incubated with 250 nM torin 1 and/or 5 µg/ml of tunicamycin for 2 h. Cell lysate extracts were assayed via Western blot analysis with antibodies against total, phospho-S6 (Ser^235/236^) and α-actinin. Data are presented as the mean ± SEM (n = 3–6 per group). **B**–**G** mRNA levels in LHCN-M2 myotubes incubated with 250 nM torin 1 and/or 5 µg/ml of tunicamycin for 8 h. Data are presented as the mean ± SEM (n = 3–6 per group). **H** PGC-1α protein levels in nuclear extracts from LHCN-M2 myotubes incubated with 250 nM torin 1 and/or 5 µg/ml of tunicamycin for 2 h and **I** in total protein extracts from the skeletal muscle of mice treated with torin 1 and/or tunicamycin for 24 h. Data are presented as the mean ± SEM (n = 5 per group). **J** TNF-α protein levels in LHCN-M2 myotubes incubated with 250 nM torin 1 and/or 5 µg/ml of tunicamycin for 16 h (n = 3–6 per group). *p < 0.05, **p < 0.01 and ***p < 0.001 versus control. ^#^*p* < 0.05, ^##^*p* < 0.01 and ^###^*p* < 0.001 versus tunicamycin

Next, we explored new potential mechanisms by which ER stress reduced PGC-1α expression in skeletal muscle. We focused on mTOR because it is activated by ER stress and inhibition of the mTOR downstream target S6 kinase (S6K) hinders ER stress-induced insulin resistance without affecting the levels of ER stress markers [18]. We used torin 1, an mTOR kinase inhibitor that inhibits both mTOR complex 1 (mTORC1) and mTORC2, to evaluate the role of mTOR in the ER stress-mediated PGC-1α reduction. Tunicamycin increased the phosphorylation levels of the ribosomal S6 subunit, a target of S6K in the mTORC1 pathway, indicating activation of the mTOR pathway. This increase was completely abrogated by torin 1 (Fig. 4A). Remarkably, mTOR inhibition by torin 1 completely prevented the reduction in *PGC-1α* expression caused by tunicamycin (Fig. 4B), suggesting the involvement of mTOR in the reduction of PGC-1α expression. However, the increase caused by tunicamycin in the mRNA levels of *ATF4* and of its target genes *CHOP* and *TRIB3* was not significantly affected by torin 1 (Fig. 4C–E). This is in line with a previous study showing that mTOR inhibition did not improve ER stress markers [18]. A different behavior was observed for *FGF21*, given that its tunicamycin-mediated induction was prevented by co-incubation with torin 1 (Fig. 4F). Since FGF21 is a marker of cellular stress, the reduction in its expression by torin 1 might reflect an amelioration of this process [29]. The effect of torin 1 was specific for *PGC-1α*, since it was not observed for *PGC-1β* (Fig. 4G). Torin 1 also prevented the reduction in PGC-1α protein levels elicited by tunicamycin in both the cultured myotubes (Fig. 4H) and mouse skeletal muscle (Fig. 4I). Collectively, these findings suggest that ER stress reduces PGC-1α expression through a new ATF4-independent PGC-1α mechanism. Moreover, in agreement with a role for PGC-1α downregulation in the exacerbation of the inflammatory response [6], the reduction in the protein levels of this transcriptional coactivator was accompanied by an increase in TNF-α protein levels in myotubes, whereas this increase was prevented by torin 1 treatment (Fig. 4J).

### mTORC1 inhibition prevents the reduction in PGC-1α and IRS1 caused by tunicamycin

**Fig. 5:**
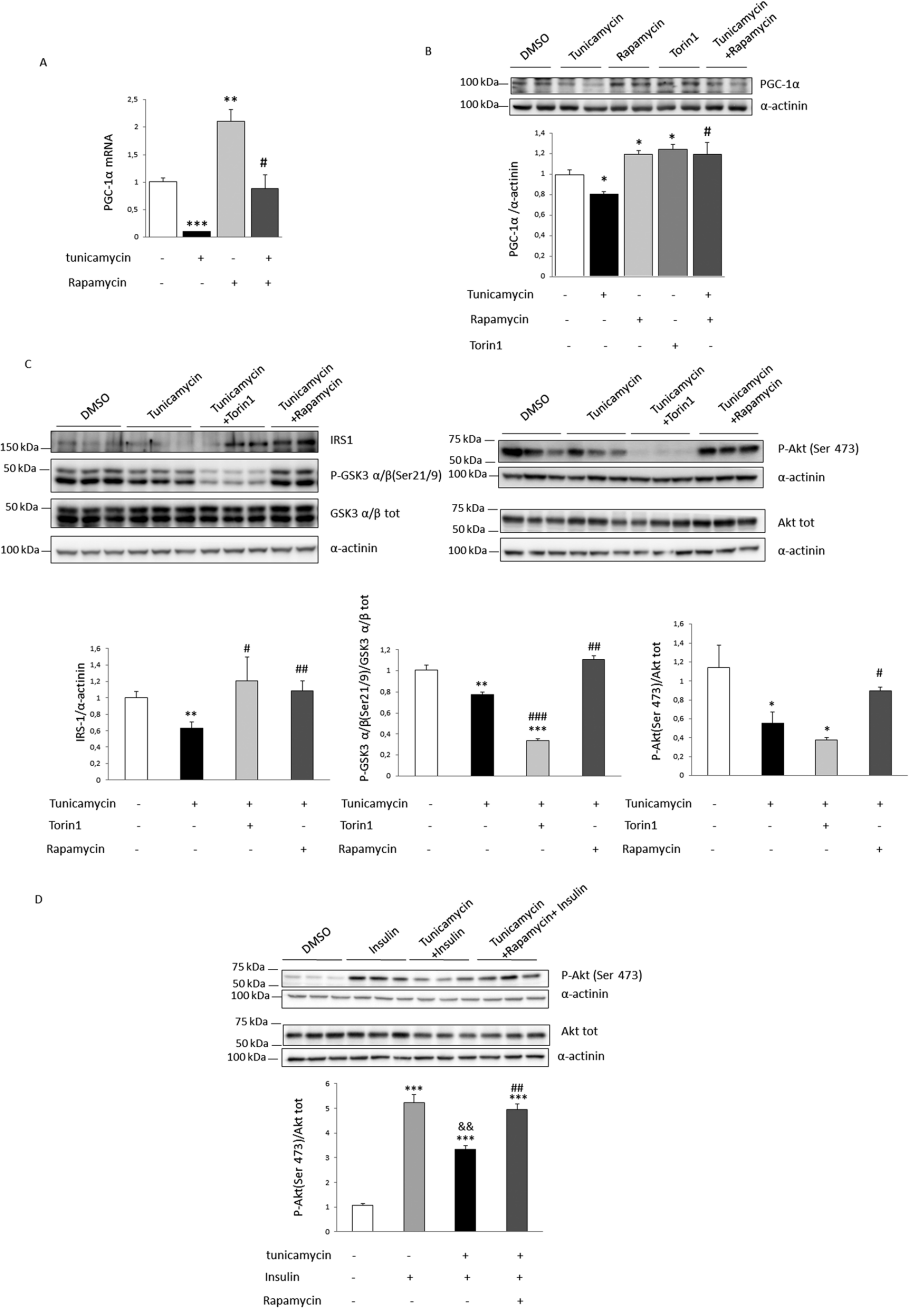
mTORC1 inhibition prevents the reductions in PGC-1α, IRS1 and the insulin signaling pathway caused by ER stress. **A** mRNA levels and **B**, **C** protein levels in human LHCN-M2 myotubes incubated with 250 nM torin1 or 100 nM rapamycin and/or 5 µg/ml of tunicamycin for 16 h (for PGC-1α, IRS1, total GSK3 α/β and phospho-GSK3 α/β (Ser21/9)) or 2 h (for total Akt and phospho-Akt (Ser 473)). **D** LHCN-M2 myotubes incubated with 100 nM rapamycin and/or 5 µg/ml of tunicamycin for 16 h with or without 100 nM of insulin (Ins) for the last 30 min. Data are presented as the mean ± SEM (n = 3–5 per group). *p < 0.05, **p < 0.01 and ***p < 0.001 versus control. ^#^*p* < 0.05, ^##^*p* < 0.01 and ^###^*p* < 0.001 versus tunicamycin. ^&&^*p* < 0.01 versus insulin-stimulated cells

Since torin 1 is a double inhibitor of mTORC1 and mTORC2, we used the selective mTORC1 inhibitor rapamycin to evaluate whether the reduction caused by tunicamycin in PGC-1α depended on this complex. Rapamycin, like torin 1, prevented the decrease in PGC-1α expression (Fig. 5A) and protein levels (Fig. 5B) caused by tunicamycin in human myotubes, indicating that this reduction involved mTORC1 activation. Because the activation of ER stress by tunicamycin also reduced IRS1 protein levels, we examined whether mTOR inhibition prevented this decrease and affected the insulin signaling pathway. Assessment of IRS1 protein levels showed that their reduction caused by tunicamycin was prevented by both torin 1 and rapamycin (Fig. 5C). Since IRS1 is crucial in insulin signaling, we next evaluated this pathway. It is well-known that torin 1 interferes with this pathway due to its inhibitory effect on mTORC2, which in turn blocks Akt phosphorylation [30]. In agreement with this, torin 1 exacerbated the reduction in the phosphorylation of Akt and its target GSK3β. However, the restoration of IRS1 caused by rapamycin in tunicamycin-exposed myotubes resulted in the recovery of the phosphorylation levels of Akt and GSK3β (Fig. 5C). Likewise, when cells were stimulated when insulin, the reduction in insulin-stimulated Akt phosphorylation caused by tunicamycin treatment was prevented by rapamycin treatment (Fig. 5D). These data indicated that tunicamycin reduces both PGC-1α and IRS1 levels and attenuates the insulin signaling pathway via mTORC1.

### mTOR/S6K inhibition prevents the ER stress-mediated downregulation of PGC-1α by restoring CRTC2 expression.

**Fig. 6:**
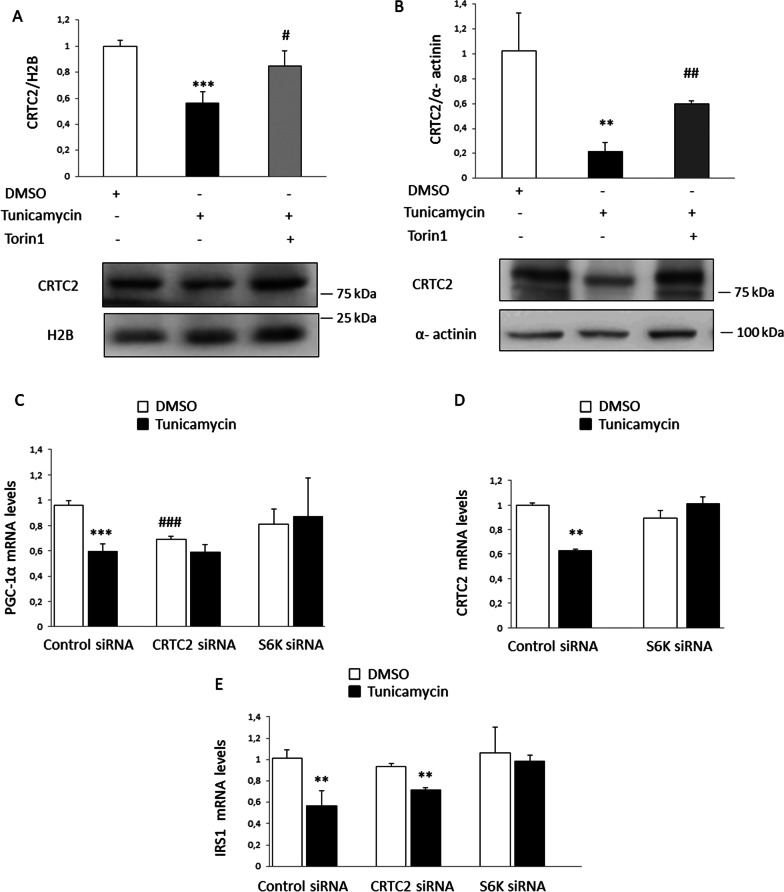
mTOR/S6K inhibition prevents the reduction of the expression of CRTC2 caused by ER stress. **A** CRTC2 protein levels in nuclear extracts from LHCN-M2 myotubes incubated with 250 nM torin 1 and/or 5 µg/ml of tunicamycin for 1 h and **B** in nuclear extracts from the skeletal muscle of mice treated with torin 1 for 24 h and/or tunicamycin for the last 8 h. **C**–**E** mRNA levels in LHCN-M2 cultured myotubes transfected with control siRNA, CRTC2 siRNA or S6K siRNA and incubated in the presence or absence (control) of 5 µg/ml of tunicamycin for 2 h. Data are presented as the mean ± SEM (n = 3–5 per group). *p < 0.05, **p < 0.01 and ***p < 0.001 versus control. ^##^*p* < 0.01 and ^##^*p* < 0.01 versus tunicamycin (**A**, **B**) or control siRNA (**C**–**E**), respectively

Given that CRTC2 is a coactivator of CREB, highly expressed in skeletal muscle and enhances CREB-dependent *PGC-1α* transcription [20] and as mTORC1 inhibition has been reported to prevent the downregulation of the CREB-target gene cyclooxygenase 2 (COX-2) in adipocytes via CRTC2 [31], we evaluated the potential role of this coactivator. CRTC2 is regulated by nuclear import [20] and nuclear levels of CRTC2 were markedly reduced in both myotubes exposed to tunicamycin (Fig. 6A) and the skeletal muscle of mice treated with this ER stressor (Fig. 6B), with these effects of tunicamycin blunted by torin 1.

To demonstrate that tunicamycin reduces PGC-1α levels by modulating CRTC2, we transfected human myotubes with different siRNAs. *CRTC2* knockdown reduced the expression of *PGC-1α* to values similar to those attained with tunicamycin treatment, confirming the key role of CRTC2 in the regulation of *PGC-1α* expression (Fig. 6C). Interestingly, knockdown of *S6K*, an mTORC1 downstream target, prevented the reduction in *PGC-1α* expression caused by tunicamycin, indicating that this kinase is involved in the ER stress-mediated reduction of the transcriptional coactivator. Likewise, *S6K* knockdown prevented the reduction in the expression levels of *CRTC2* caused by tunicamycin (Fig. 6D). Together, these findings indicated that attenuation of the mTOR/S6K pathway prevents the decrease in CRTC2 and the subsequent reduction in PGC-1α caused by tunicamycin.

By contrast, CRTC2 knockdown did not affect basal *IRS1* mRNA levels (Fig. 6E), indicating that this gene is not under the control of this coactivator in human myotubes. However, *S6K* knockdown prevented the reduction in *IRS1* expression caused by tunicamycin. Thus, *IRS1* downregulation by tunicamycin involves the mTORC1/S6K pathway, independently of CRTC2.

## Discussion

PGC-1α downregulation in skeletal muscle contributes to insulin resistance and T2DM [12]. Therefore, the study of the mechanisms underlying decreases in PGC-1α reduction in skeletal muscle might bring to light the pathways involved in insulin resistance development. Here, we show two new mechanisms through which ER stress reduced PGC-1α levels in skeletal muscle. First, increased expression of the transcription factor ATF4 mediated the reduction in PGC-1α caused by either palmitate- or tunicamycin-induced ER stress. The findings of this work demonstrated that ATF4 overexpression decreased basal PCG-1α expression, whereas ATF4 knockdown abolished the reduction of PCG-1α caused by tunicamycin, but only attenuated the effect of palmitate on this transcriptional coactivator. The lower effect of ATF4 knockdown in cells exposed to palmitate indicates that this fatty acid can also reduce PGC-1α by another mechanism, as we have previously reported [6].

It has been previously reported that during the regulation of responses to cold stress in brown adipocytes, ATF4 acts as a negative regulator of PGC-1α by competing with CREB for the binding to the CRE site in the *Pgc-1α* promoter [14]. It is likely that this same mechanism operates in skeletal muscle following ER stress. In agreement with the decrease in PGC-1α, the expression of PPARα, which is co-activated by PGC-1α, and their target genes involved in fatty acid oxidation is reduced alongside palmitate oxidation. As a result of these changes, fatty acid-induced ER stress might reduce the catabolism of this lipid, exacerbating the effects of palmitate on ER stress as well as on the synthesis of palmitate-derived deleterious complex lipids that promote the development of inflammation and insulin resistance.

Second, we show an additional new mechanism involved in the reduction of PGC-1α expression in skeletal muscle that connects ER stress to mTOR. Based on our findings, we propose that ER stress activates mTOR in myotubes, which in turn reduces the nuclear levels of CRTC2. CRTC2 is a CREB coactivator that is highly expressed in skeletal muscle. It is regulated by nuclear import and greatly enhances CREB-dependent PGC-1α transcription [20]. In fact, family members of CRTC2 have been described to be the most potent activators of PGC-1α transcription [20]. In fasting states, nuclear CRTC2 promotes hepatic gluconeogenesis by inducing the expression of PGC-1α [32]. The role of CRTC2 in the reduction of PGC-1α expression caused by ER stress was unknown. In this study, we observed that CRTC2 knockdown in human myotubes resulted in a decrease in PGC-1α expression, confirming the role of CRTC2 in the regulation of PGC-1α in our cell model. In addition, our findings showed that mTOR inhibition abolished the reduction in the PGC-1α mRNA levels in myotubes caused by the ER stressor tunicamycin. This was accompanied by a recovery in the nuclear CRTC2 levels. This reduction in CRTC2 is likely to involve a transcriptional mechanism, since knockdown *S6K*, an mTORC1 downstream target, prevented the decrease in *CRTC2* mRNA levels caused by tunicamycin. A previous study reported that adipose mTOR activation also reduced the expression of COX-2, a target of CREB, by regulating CRTC2 [31]. However, that study demonstrated that mTOR activation resulted in the phosphorylation of CRTC2 at Ser^136^, which inhibited binding of CREB to the *Cox-2* promoter. Furthermore, rapamycin prevented the phosphorylation of CRTC2 at Ser^136^ without affecting the nuclear translocation of CRTC2. Another study reported that hepatic mTOR activation in obesity led to the phosphorylation of cytosolic CRTC2 at Ser^136^, which in turn elicited SREBP1 processing and increased hepatic lipogenesis without any apparent changes in CRTC2 cell localization [33]. That study also showed that CRTC2 phosphorylation at Ser^136^ was inhibited by mTOR inhibitors, with this inhibition more sensitive to torin 1 than to rapamycin, suggesting a more important role for mTORC2. We do not know the reasons for the discrepancies between the other studies and ours regarding the effects of mTOR on nuclear CRTC2 levels, but differences in the cell types used might be one explanation. In fact, differences in the regulation of CRTC2 between the liver and skeletal muscle might be due to different functions, similar to what has been described for PGC-1α. This transcriptional coactivator stimulates gluconeogenesis in the liver, favoring hyperglycemia, whereas it improves insulin sensitivity in skeletal muscle.

The studies published so far indicate that the mTOR-CRTC2 pathway in the liver and adipose tissue contributes to insulin resistance development. Thus, mTOR hyperactivation by overnutrition results in an increase in hepatic CRTC2 phosphorylation at Ser^136^ that promotes lipogenesis [33]. Moreover, HFD-fed CRTC2^−/−^ mice show decreased plasma glucose levels and improved insulin sensitivity, partly through the effects on hepatic gluconeogenesis [34]. Likewise, mTOR activation inhibits white adipocyte browning by inhibiting the COX-2-mediated synthesis of prostaglandins [31]. In contrast to these effects, our findings demonstrated that the activation of mTOR/S6K signaling by ER stress in skeletal muscle resulted in a decrease in CRTC2 levels, leading to a subsequent reduction in PGC-1α expression, thereby contributing to the development of insulin resistance. It remains to be seen how S6K reduces CRTC2 protein levels in myotubes. Thus, these data show a different role for the mTOR-CRTC2 axis in skeletal muscle, to that previously reported for the liver, which is consistent with the effects of PGC-1α, a direct target of CRTC2, in this tissue. Finally, although it has been reported that mTORC1 activation results in ATF4 phosphorylation and the subsequent increase in purine synthesis [35], torin 1 did not affect ATF4 expression in our conditions, suggesting that the effect of mTOR activation on PGC-1α expression does not rely on ATF4.

Overnutrition augments mTORC1 signaling, which in turn may generate insulin resistance via mTORC1/S6K-mediated IRS1 serine phosphorylation as well as the degradation of its total protein levels, attenuating the insulin signaling pathway [36, 37]. However, IRS1 transcriptional regulation has been poorly studied and the role of ER stress in the expression of this gene is unknown. Here, we also demonstrated that the induction of ER stress reduced IRS1 mRNA levels in skeletal muscle via mTOR/S6K. Interestingly, mTORC1 inhibition by rapamycin restored IRS1 mRNA levels and phosphorylated Akt levels in basal and insulin-stimulated conditions, indicating that reduced IRS1 transcription during ER stress contributes to the attenuation of the insulin signaling pathway, with this pathway recovering upon mTOR/S6 inhibition.

## Conclusions

Overall, our findings indicate that the activation of the mTOR/S6K axis by ER stress results in a reduction in PGC-1α expression in myotubes via CRTC2 downregulation, confirming that targeting the mTOR/S6K axis in diabetic states may improve insulin sensitivity in skeletal muscle by preventing the decreases in PGC-1α and IRS1 levels.

## Supplementary Information

**Additional file 1:** mRNA levels following overexpression and siRNA transfection.

## Data Availability

Not applicable.

## Abbreviations

ACADM: Acyl-CoA dehydrogenase medium chain
ACOX: Acyl-CoA oxidase
AMPK: AMP-activated protein kinase
ATF4: Activating transcription factor 4
CHOP: C/EBP homologous protein
CPT-1: Carnitine palmitoyl-transferase 1
CREB: CAMP response element-binding protein
CRTC2: CREB-regulated transcription coactivator 2 (also known as transducer of regulated CREB activity 2; TORC2)
eIF2α: Eukaryotic translation initiation factor 2α
ER: Endoplasmic reticulum
ERK: Extracellular signal–regulated kinase
FGF21: Fibroblast growth factor 21
HFD: High-fat diet
IRS: Insulin receptor substrate
mTOR: Mammalian target of rapamycin
PBA: 4-Phenylbutyric acid
PERK: Protein kinase RNA-like ER kinase
PGC-1α: PPARγ coactivator 1α
PPAR: Peroxisome proliferator-activated receptor
TRIB3: Tribbles 3
UPR: Unfolded protein response
VLDLR: Very low density lipoprotein receptor
