# Supplementary material for: Endoplasmic reticulum stress downregulates PGC-1α in skeletal muscle through ATF4 and an mTOR-mediated reduction of CRTC2
Source: Cell Commun Signal. 2022 Apr 15;20:53. doi: 10.1186/s12964-022-00865-9 (PMC9012021; doi:10.1186/s12964-022-00865-9)
Supplement: Supplementary file 2 — Additional file 1: mRNA levels following overexpression and siRNA transfection. [file 12964_2022_865_MOESM2_ESM.pdf]

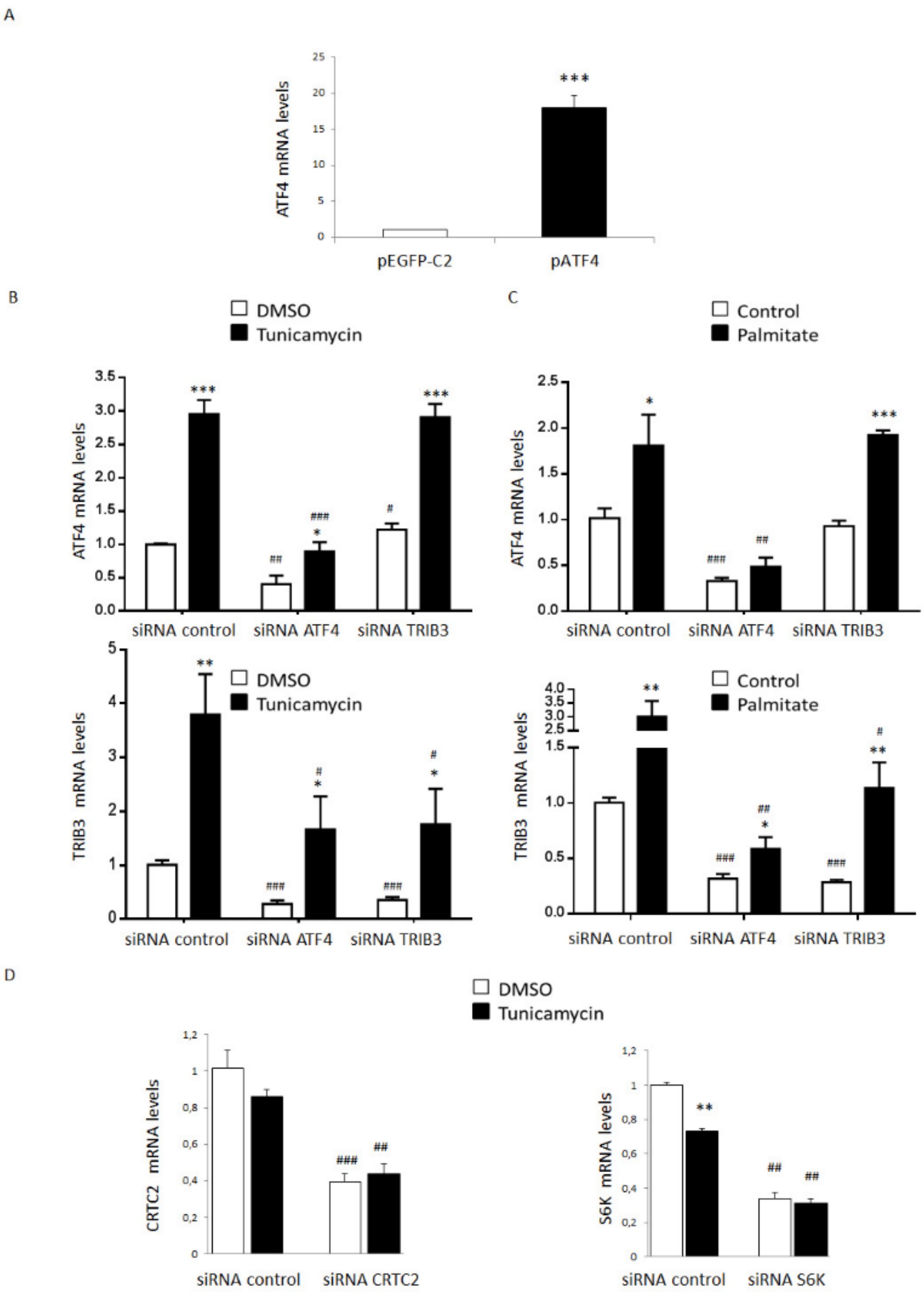

Supplementary Figure 1. (A) ATF4 mRNA levels of LHCN-M2 cultured myotubes transfected with pCMV-EGFP-C2 (pEGFP-C2) or pCMV-ATF4 (pATF4). Data are presented as the mean ± SEM (n = 6 per group). ATF4 and TRIB3 mRNA levels in LHCN-M2 cultured myotubes transfected with control siRNA, ATF4 siRNA or TRIB3 siRNA and incubated in the presence or absence (control) of (B) 2 µg/ml of tunicamycin or (C) 0.5 mM palmitate for 16 h. Data are presented as the mean ± SEM (n = 4 per group). (D) CRTC2 and S6K mRNA levels in LHCN-M2 cultured myotubes transfected with control siRNA, CRTC2 siRNA or S6K siRNA and incubated in the presence or absence (control) of 5 µg/ml of tunicamycin for 2 h. Data are presented as the mean ± SEM (n = 3-5 per group). *p < 0.05, **p < 0.01 and ***p < 0.001 *vs.* control. $^{\#}p < 0.05$, $^{\#\#}p < 0.01$, and $^{\#\#\#}p < 0.001$ *vs.* tunicamycin or control siRNA.
